# Editorial Comment: Fluid Intake and Dietary Factors and the Risk of Incident Kidney Stones in UK Biobank: A Population-based Prospective Cohort Study

**DOI:** 10.1590/S1677-5538.IBJU.2021.04.04

**Published:** 2021-07-20

**Authors:** Fábio C. M. Torricelli

**Affiliations:** 1 Hospital das Clínicas da Faculdade de Medicina da Universidade de São Paulo Divisão de Urologia do Hospital das Clínicas São PauloSP Brasil Divisão de Urologia do Hospital das Clínicas da Faculdade de Medicina da Universidade de São Paulo. São Paulo, SP, Brasil.

Thomas J. Littlejohns 1, Naomi L. Neal 2, Kathryn E. Bradbury 3, Hendrik Heers 4, Naomi E. Allen 5, Ben W. Turney 6

Eur Urol Focus. 2020 Jul 15;6(4):752-761

## COMMENT

Prevalence of kidney stone disease is high and it is still increasing (1). Stone recurrence rate is also high and can achieve half of patients in a 10-year follow-up (2). Therefore, nephrolithiasis has a significant impact on kidney stone formers life and important costs related to patient care. In this scenario, prevention work is an essential tool to decrease patient's suffering and reduce medical expenses.

Fluids intake, vegetables and fruits consumption and some herbs are considered protective against kidney stone formation, whereas salt and meat are associated with a higher chance of nephrolithiasis (3–5). However, most of studies have limited number of patients and some data are conflicting. Littlejohns et al have presented a study with a large population from a prospective database including almost 500 000 people showing that higher total fluid intake is associated wit a lower risk of kidney stones after adjustment for lifestyle and socioecomic factors. Also fruits and fibers are protective, while meat and salt are associated with a increased risk of kidney stone formation. Although these findings are not really a novelty, this study provides a better quality evidence for our recommendations when clinically managing kidney stone formers. Of course, this study has limitations inherent to its design, as it does not take into account dietary changes overtime and some potential confounders may not be considered in the analysis.

Urologists should be encouraged to counsel their patients for dietary modifications aiming to reduce kidney stone formation. It is a low cost strategy and easy method for improving patient's quality of life.
